# The impact of current smoking, regular drinking, and physical inactivity on health care-seeking behavior in China

**DOI:** 10.1186/s12913-022-07462-z

**Published:** 2022-01-10

**Authors:** Changle Li, Jing Sun

**Affiliations:** School of Health Management, Inner Mongolia Medical University, Hohhot, 010110 China

**Keywords:** Lifestyle, Smoking, Regular drinking, Physical inactivity, Health care-seeking behavior

## Abstract

**Background:**

People with lifestyle behaviors, such as current smoking, regular drinking, and physical inactivity, may experience a lack of or delayed health care, leading to severe sickness and higher health care expenditures in the future. Hence, the current study aims to ascertain the effects of current smoking, regular drinking, and physical inactivity on health care-seeking behavior among adults who report physical discomfort in China.

**Methods:**

The data used in this study were obtained from the China Family Panel Studies (CFPS). The final sample consisted of 44,362 individuals who participated in all five waves of data collection. Logistic regression models were used for the analysis.

**Results:**

The results of fixed effects logistic regression showed that among those who reported physical discomfort, adults who currently smoked cigarettes were 0.65 times less likely to seek health care than those who formerly smoked. Compared to nondrinkers, adults who regularly drank alcohol had a decreased likelihood of seeking health care. Adults who never engaged in physical exercise had 24% lower odds of seeking health care than those who engaged in physical exercise.

**Conclusions:**

Current smoking, regular drinking, and physical inactivity decreased the probability of seeking health care among adults who reported physical discomfort. Therefore, screening and brief advice programs should be delivered by primary-level care and should pay more attention to individuals who engage in lifestyle behaviors such as current smoking, regular drinking, and physical inactivity, thus avoiding missed opportunities to treat chronic conditions and detect new diseases early.

## Background

In 2015, a Chinese national nutrition and chronic disease report indicated that the prevalences of current smoking, harmful drinking, and physical inactivity among adults were 28.1, 9.3, and 71.3%, respectively [1]; these preventable risk factors have contributed to the increased rise in chronic diseases. Chronic diseases now account for approximately 80% of total deaths and 70% of total disability-adjusted life years lost in China [2].

Lifestyles have proven to be independent or synergistical causes of diseases, such as hypertension, dyslipidemia, diabetes, and obesity [3]. Most people make lifestyle changes due to the health concerns and illnesses they experience. For example, in 2018, 38.7% of Chinese smokers stopped smoking because they were worried about their future health status, and 26.6% stopped smoking because they experienced severe sickness [4]. In addition, former smoking has been associated with higher health care utilization and increased health care expenditures [5–8]. People with lifestyle behaviors including current smoking, regular drinking, and physical inactivity may not care about their health status or may be risk-tolerant individuals. As a result, they may experience a lack of or delayed health care, thereby leading to severe sickness and higher health care expenditures in the future [9–11].

To our knowledge, little is known about the association between lifestyle factors and health care-seeking behavior [12, 13]. Hence, the current study aims to ascertain the effects of current smoking, regular drinking, and physical inactivity on health care-seeking behavior among adults who report physical discomfort in China. This knowledge will help better understand the associations between current smoking, regular drinking, and physical inactivity and health care-seeking behavior, thus helping the government make health care resource allocation decisions and improve health education programs for target populations.

## Methods

### Data source and study sample

The data used in this study were obtained from the China Family Panel Studies (CFPS), conducted by the Institute of Social Science Survey of Peking University. The CFPS is a nationally representative, biennial survey designed to collect Chinese community-, family-, and individual-level longitudinal data; the data cover twenty-five provinces and their administrative equivalents, representing approximately 95% of the total population in mainland China. The CFPS uses multistage probability proportional-to-size sampling. More details of the data collection process were given in Xie and Lu [14]. The CFPS questionnaires include questions on demographic background, family structure/transfer, health status and physical functioning, health care utilization, insurance status, work, income, expenditure, asset ownership, community-level information, etc. [15].

The CFPS primarily conducts face-to-face interviews. Telephone or online interviews are used as a substitute only when the CFPS fails to complete face-to-face interviews. In the first wave in 2010, 14,960 households, including 33,600 adults (above 16 years old) and 8990 children, were successfully interviewed; in four waves of full sample follow-up surveys in 2012, 2014, 2016, and 2018, 13,315 households (35,720 adults and 8624 children), 13,946 households (37,147 adults and 8617 children), 14,019 households (36,892 adults and 8427 children), and 14,218 households (37,354 adults and 8735 children) were successfully interviewed, respectively. More details about the CFPS are available from Xie and Hu [16].

From the full sample, only the adults (16 years old or older) who reported feeling any physical discomfort in the past 2 weeks prior to the survey interview were selected. The final sample consisted of 44,362 individuals who participated in all five waves of data collection.

### Measures

#### Dependent variable

Health care-seeking behavior was set as a binary variable, indicating the decision to consult a doctor (or not) among the adults who reported feeling any physical discomfort in the past 2 weeks. The question in the CFPS that represent this variable is: ‘Have you seen a doctor within the past two weeks?’. Response options are: Yes or no.

#### Independent variables

The respondents completed a face-to-face interview about lifestyle factors, including current smoking, regular drinking, and physical inactivity. In the CFPS, each respondent was asked, ‘Have you smoked in the past month?’. A respondent reporting ‘Yes’ was categorized as current smoking. Respondents who reported ‘No’ were then asked, ‘Have you ever smoked? Yes or No’. If the respondent reported ‘Yes’, the respondent was categorized as former smoking. If the respondent reported ‘No’ to both questions, the respondent was categorized as never smoking. Regular drinking was defined as a binary variable. The CFPS question supporting this variable was ‘Have you often drunk alcohol (at least three times a week) in the past month?’. Respondents reporting ‘Yes’ were coded as 1, and those reporting ‘No’ were coded as 0. In the CFPS, the respondents were asked how often they participated in physical exercise in the past week. The respondents were categorized as physical inactivity if they answered “Never”.

The other independent variables were selected based on previous studies and included the respondent’s age, gender, marital status, urban residency, household income, medical insurance status, educational attainment, employment status, self-reported health status, chronic conditions, and severity of physical discomfort (SPD). A single question was used to measured SPD, which was classified into three categories by asking the respondents how serious they thought their physical discomfort was: mild, moderate, or serious. Definitions of all relevant variables are provided in Table 1.

**Table 1:** Definitions of variables

Variable	Description
**Dependent variable**	
Health care-seeking behavior	Coded: 1 if the individual self-reported consulting a doctor; 0 otherwise
**Independent variable**	
Smoking status	
Current smoking	Coded: 1 if the individual currently smokes cigarettes; 0 otherwise
Former smoking	Coded: 1 if the individual has quit smoking; 0 otherwise
Never smoking	Coded: 1 if the individual has never smoked; 0 otherwise
Regular drinking	Coded: 1 if the individual drank alcohol at least 3 times a week in past month; 0 otherwise
Physical inactivity	Coded: 1 if the individual never participated in physical exercise in the past week; 0 otherwise
Age group	
16–24	Coded: 1 if the individual is 16–24 years old; 0 otherwise
25–64	Coded: 1 if the individual is 25–64 years old; 0 otherwise
> =65	Coded: 1 if the individual is > = 65 years old; 0 otherwise
Male	Coded: 1 if the individual is male; 0 for female
Educational attainment	
Illiterate	Coded: 1 if the individual is illiterate; 0 otherwise
Elementary school	Coded: 1 if the individual attends elementary school; 0 otherwise
Middle school	Coded: 1 if the individual graduated from middle school; 0 otherwise
High school	Coded: 1 if the individual graduated from high school; 0 otherwise
Above three-year college	Coded: 1 if the individual graduated from above three-year college; 0 otherwise
Married	Coded: 1 if the individual is married; 0 otherwise
Urban residency	Coded: 1 if the individual is an urban resident; 0 for rural resident
Medical insurance	
GMI	Coded: 1 if the individual is enrolled in the Government Medical Insurance; 0 otherwise
UEMI	Coded: 1 if the individual is enrolled in the Urban Employee Medical Insurance; 0 otherwise
URMI	Coded: 1 if the individual is enrolled in the Urban Resident Medical Insurance; 0 otherwise
NRCMI	Coded: 1 if the individual is enrolled in the New Rural Cooperative Medical Insurance; 0 otherwise
Other Insurance	Coded: 1 if the individual is enrolled in supplementary medical insurance; 0 otherwise
No Insurance	Coded: 1 if the individual does not have medical insurance; 0 otherwise
Household income	Net household income (10,000 Yuan)
Economically active	Coded: 1 if the individual reports participating in agricultural jobs, working for wages for an employer, or working for oneself rather than an employer; 0 if the individual reports being a temporary worker, retired, unemployed, or a student;
Health status	
Poor	Coded: 1 if the individual reports his or her health status to be poor; 0 otherwise
Fair	Coded: 1 if the individual reports his or her health status to be fair; 0 otherwise
Good	Coded: 1 if the individual reports his or her health status to be good, very good, or excellent; 0 otherwise
Chronic conditions	Coded: 1 if the individual has had doctor-diagnosed chronic diseases in the past 6 months; 0 otherwise
Severity of physical discomfort	
Mild	Coded: 1 if the individual perceives himself or herself to have had mild physical discomfort in the past 2 weeks; 0 otherwise
Moderate	Coded: 1 if the individual perceives himself or herself to have had moderate physical discomfort in the past 2 weeks; 0 otherwise
Serious	Coded: 1 if the individual perceives himself or herself to have had serious physical discomfort in the past 2 weeks; 0 otherwise

### Statistical analysis

Bivariate analyses were used to examine differences between adults who consulted a doctor (seeking health care) and those who did not consult a doctor when they felt physical discomfort in each wave. Pearson’s chi-square test was used to analyze the categorical independent variables.

The current study examined the relationship between the dependent variable and independent variables using pooled and panel data estimation. Pooled estimation can increase the sample size, but it is often biased or at least inefficient [17]. Therefore, a pooled regression model can be a starting point. After the use of the pooled regression model, the data were treated as having a panel structure, and a choice between fixed and random effects models had to be made. The panel structure can increase the degrees of freedom and decrease the collinearity among independent variables. Based on Hausman’s specification test, fixed effects estimation should be preferred over random effects estimation. However, when estimating the fixed effects model, many pieces of information are lost. Therefore, a random effects model was also presented in this study [17, 18].

Since the dependent variable was a binary response variable, logistic regression was used to examine the impact of current smoking, regular drinking, and physical inactivity on health care-seeking behavior in China. The final model was adjusted for all the confounding variables. The results are presented as odds ratios (ORs) along with 95% confidence intervals (CIs). All statistical analyses were carried out using the statistical software package STATA 15.

## Results

A descriptive summary of all variables over time is shown in Table 2. Among those who reported physical discomfort, the proportion of people seeking health care increased from 68.8% in 2010 to 76.1% in 2018. Approximately 26.0% of adults currently smoked tobacco products from 2010 to 2018. Approximately 12.0% of adults regularly drank alcohol from 2010 to 2018. The prevalence of physical inactivity was 72.0% in 2010. This proportion decreased to 52.8% in 2018.

**Table 2:** Description of the selected variables in the five waves (percentage)

	2010 *N* = 8358	2012 *N* = 9061	2014 *N* = 8774	2016 *N* = 9021	2018 *N* = 9148
Health care-seeking behavior	68.8	65.4	72.3	74.9	76.1
Current smoking	28.8	27.4	26.2	24.9	25.7
Former smoking	7.3	5.0	5.2	5.2	8.2
Never smoking	63.9	67.5	68.6	69.8	66.2
Regular drinking	12.9	13.2	13.1	11.7	12.5
Physical inactivity	72.0	56.9	63.2	58.8	52.8
Age group					
16–24	6.8	7.4	6.4	7.3	6.4
25–64	76.0	74.5	73.7	70.9	69.9
> =65	17.2	18.2	20.0	21.8	23.7
Male	42.7	43.4	42.3	42.0	42.8
Educational attainment					
Illiteracy	36.1	35.4	35.4	34.5	30.4
Elementary school	21.5	21.6	22.2	21.5	21.6
Middle school	25.0	23.8	24.4	24.4	26.1
High school	11.8	12.4	11.7	11.7	12.5
Above three-year college	5.7	6.8	6.3	8.0	9.5
Married	82.5	82.5	82.3	80.4	79.9
Urban residency	44.8	45.4	47.1	47.5	47.7
Medical insurance					
GMI	4.7	3.7	2.9	1.9	2.4
UEMI	9.5	12.2	12.6	12.4	13.1
URMI	7.0	7.6	8.2	8.0	7.9
NRCMI	58.1	64.6	67.5	68.2	67.7
Other Insurance	0.5	0.3	0.6	0.4	0.3
No Insurance	20.2	11.7	8.2	9.1	8.6
Household income ^a^	3.3 (5.3)	4.2 (5.0)	4.6 (6.1)	5.4 (12.5)	5.6 (8.5)
Economically active	45.4	67.5	71.5	75.3	73.2
Health status					
Poor	38.9	41.0	36.1	36.2	36.4
Fair	42.0	23.0	19.5	22.6	16.7
Good	19.1	36.0	44.4	41.3	46.9
Chronic conditions	29.0	22.1	34.6	34.0	32.7
Severity of physical discomfort					
Mild	22.2	24.1	20.8	20.9	17.3
Moderate	40.1	39.7	47.2	42.3	44.4
Serious	37.7	36.2	32.0	36.8	38.4

RMB: 1000 Chinese Renminbi is approximately 150 US$

^a^Values are expressed as the mean (standard deviation)

Table 3 compares adults who sought health care and those who did not seek health care when they felt physical discomfort based on a variety of lifestyle factors. Among those who reported physical discomfort, adults who currently smoked were less likely to seek health care, and a similar trend was observed for adults who regularly drank alcohol. In addition, the chi-square test found that current smoking and regular drinking were significantly associated with health care-seeking behavior in some or all five waves.

**Table 3:** Lifestyle behaviors of adults who sought health care and those who did not seek health care

	2010		2012		2014		2016		2018	
	*N* = 8358		*N* = 9061		*N* = 8774		*N* = 9021		*N* = 9148	
	Seeking health care		Seeking health care		Seeking health care		Seeking health care		Seeking health care	
	Yes	No	Yes	No	Yes	No	Yes	No	Yes	No
Current smoking (%)										
Yes	67.7	32.3	61.3	38.7	69.6	30.4	72.7	27.3	73.1	26.9
No	69.2	30.8	67.0	33.0	73.3	26.7	75.7	24.3	77.1	22.9
*P* value	0.185		< 0.001		0.001		0.005		< 0.001	
Regular drinking (%)										
Yes	62.3	37.7	57.0	43.1	64.9	35.1	69.7	30.3	69.8	30.2
No	69.7	30.3	66.7	33.3	73.4	26.6	75.6	24.4	77.0	23.1
*P* value	< 0.001		< 0.001		< 0.001		< 0.001		< 0.001	
Physical inactivity (%)										
Yes	68.7	31.3	65.5	34.5	71.8	28.2	74.2	25.8	74.2	25.8
No	69.0	31.0	65.3	34.8	73.1	26.9	76.0	24.0	78.1	21.9
*P* value	0.795		0.778		0.204		0.045		< 0.001	

Table 4 presents the results of the regression analysis for the pooled logistic, random effects logistic, and fixed effects logistic models. The likelihood ratio test and Hausman’s specification test showed highly significant test statistics (LR = 569.63 and *χ*^2^(24) =162.32). These results further demonstrated that the fixed effects logistic model should be preferred over the random effects logistic model. The results of the logistic regression analysis are shown in Table 4 as ORs. An OR greater than one indicated a positive effect on the likelihood of seeking health care, while an OR less than one indicated a negative effect.

**Table 4:** Logistic regression analysis of health care-seeking behavior

	Pooled logistic	Random effects logistic	Fixed effects logistic
	(i)	(ii)	(iii)
	Odds Ratios (95% CI)	Odds Ratios (95% CI)	Odds Ratios (95% CI)
Smoking status			
Current smoking	0.84^***^ (0.75, 0.93)	0.79^***^ (0.69, 0.89)	0.65^***^ (0.50, 0.84)
Never smoking	0.90^*^ (0.80, 1.00)	0.86^**^ (0.75, 0.98)	0.79^*^ (0.60, 1.04)
Former smoking (ref.)			
Regular drinking	0.76^***^ (0.71, 0.82)	0.72^***^ (0.67, 0.79)	0.77^***^ (0.65, 0.91)
Physical inactivity	0.76^***^ (0.72, 0.79)	0.73^***^ (0.69, 0.77)	0.76^***^ (0.69, 0.83)
Age group			
16–24 (ref.)			
25–64	1.18^***^ (1.07, 1.31)	1.23^***^ (1.09, 1.39)	0.97 (0.69, 1.37)
> =65	1.57^***^ (1.40, 1.75)	1.74^***^ (1.52, 2.00)	1.74^***^ (1.17, 2.59)
Male	0.98 (0.92, 1.05)	0.97 (0.90, 1.06)	0.99 (0.32, 3.26)
Educational attainment			
Illiteracy (ref.)			
Elementary school	0.99 (0.93, 1.07)	1.01 (0.93, 1.10)	1.15 (0.86, 1.55)
Middle school	0.98 (0.91, 1.06)	0.99 (0.91, 1.08)	1.23 (0.83,1.81)
High school	0.79^***^ (0.73, 0.86)	0.76^***^ (0.68, 0.84)	1.18 (0.71, 1.96)
Above three-year college	0.59^***^ (0.53, 0.66)	0.53^***^ (0.47, 0.61)	1.21 (0.67, 2.20)
Married	1.24^***^ (1.16, 1.33)	1.30^***^ (1.19, 1.41)	0.91 (0.72, 1.16)
Urban residency	0.83^***^ (0.79, 0.88)	0.80^***^ (0.75, 0.86)	1.25^**^ (1.00, 1.56)
Medical insurance			
GMI	1.24^***^ (1.08, 1.43)	1.25^**^ (1.05, 1.48)	1.01 (0.74, .139)
UEMI	1.14^***^ (1.04, 1.26)	1.14^**^ (1.01, 1.27)	1.08 (0.87, 1.35)
URMI	1.14^**^ (1.03, 1.27)	1.17^**^ (1.03, 1.32)	1.19 (0.97, 1.46)
NRCMI	1.51^***^ (1.40, 1.62)	1.57^***^ (1.44, 1.71)	1.09 (0.93, 1.28)
Other Insurance	0.92 (0.67, 1.27)	0.87 (0.60, 1.27)	0.89 (0.51, 1.54)
No Insurance (ref.)			
Household income	1.01^**^ (1.00, 1.01)	1.01^***^ (1.00, 1.01)	1.01^**^ (1.00, 1.02)
Economically active	1.01 (0.96, 1.07)	1.03 (0.97, 1.10)	1.04 (0.92, 1.17)
Health status			
Poor	1.38^***^ (1.29, 1.47)	1.46^***^ (1.36, 1.58)	1.42^***^ (1.26, 1.59)
Fair (ref.)			
Good	0.94^**^ (0.89, 0.99)	0.94^*^ (0.88, 1.00)	1.04 (0.93, 1.16)
Chronic conditions	2.42^***^ (2.29, 2.57)	2.69^***^ (2.51, 2.88)	2.04^***^ (1.85, 2.25)
Severity of physical discomfort			
Mild	0.58^***^ (0.55, 0.61)	0.52^***^ (0.48, 0.55)	0.52^***^ (0.47, 0.58)
Moderate (ref.)			
Serious	1.87^***^ (1.76, 1.98)	2.09^***^ (1.95, 2.24)	1.94^***^ (1.75, 2.15)
Constant	1.44^***^ (1.21, 1.72)	1.62^***^ (1.32, 1.99)	
Observations	44,362	44,362	13,036

Likelihood ratio test in random effects logistic model: *LR* = 569.63, *p* < 0.001

Hausman’s specification test is significant at the 1% level: *χ*^2^(24) = 162.32, *p* < 0.001

Asterisks^***^ indicate statistical significance at the 1% level, ^**^ at the 5% level, and ^*^ at the 10% level

Column (iii) of Table 4 presents factors affecting health care-seeking behavior using the fixed effects logistic model. Among those who reported physical discomfort, adults who currently smoked cigarettes were 0.65 times less likely to seek health care than those who formerly smoked (OR = 0.65, 95% CI: 0.50, 0.84). Compared to nondrinkers, adults who regularly drank alcohol had a decreased likelihood of seeking health care (OR = 0.77, 95% CI: 0.65, 0.91). Adults who never engaged in physical exercise had 24% lower odds of seeking healthcare than those who engaged in physical exercise (OR = 0.76, 95% CI: 0.69, 0.83).

Irrespective of the estimation method, adults who currently smoked cigarettes, regularly drank alcohol, and never engaged in physical exercise were less likely to seek health care when they felt physical discomfort (see Columns (i)- (iii) of Table 4).

## Discussion

The current study examined the association between lifestyle factors and health care-seeking behavior among a Chinese adult general population using a five-wave longitudinal dataset. This study found that the proportion of individuals seeking health care increased from 68.8% in 2010 to 76.1% in 2018. This result is not unexpected. Since the launch of the new health reform of 2009, the Chinese government has made considerable investments in strengthening the accessibility and availability of health care facilities. As a result, almost every community (village) has at least one primary care facility. Moreover, China successfully achieved near-universal health insurance coverage, with 95% of the population covered in 2011, and proposed a zero-profit drug policy, compelling public hospitals nationwide to sell drugs at cost without mark-up fees to make health care facilities more attractive to patients [19–21].

On the other hand, the proportion of people seeking health care in China is much higher than the findings in European studies. Elnegaard et al. [22] found that the proportion of the adult Danish population contacting a general practitioner with at least one symptom was 37%. Elliott, McAteer, and Hannaford [23] reported that 20% of respondents with symptoms consulted with a primary care health professional in the UK. Health care-seeking behavior in different countries may have been affected by degrees of access to care and levels of out-of-pocket payments. Both Demark and the UK have easy and free access to health care facilities. The gap in the proportion of people seeking health care may be because the formal referral system in China is now virtually absent. Patients have the freedom to choose any health care facilities they prefer to visit, regardless of their disease severity [24]. However, people with lifestyle behaviors including current smoking, regular drinking, and physical inactivity had a lower proportion of seeking health care in China.

A fixed effects logistic regression model was used to identify the lifestyle factors affecting health care-seeking behavior. The results indicate that among those who reported physical discomfort, adults who currently smoked cigarettes, regularly drank alcohol, and never engaged in physical exercise were less likely to seek health care. Similar results have been discovered in China, England, and Australia. For example, Zhou et al. [25] observed that physical inactivity decreased the probability of seeking health care in China. Smith et al. [12] found that smokers were less likely to seek help than nonsmokers in England. Feng et al. [13] revealed that people with multiple lifestyle factors, including smoking, alcohol consumption, diet and physical inactivity, were less likely to see general practitioners in Australia.

Four possible reasons may explain the inverse relationship between lifestyle factors and health care-seeking behavior. First, people with lifestyle behaviors including current smoking, regular drinking, and physical inactivity are more risk tolerant, and they may more willingly bear disease risk [26, 27]. A higher willingness to bear risk decreases the probability of seeking health care [28]. Second, lifestyle behaviors such as smoking, drinking, and physical inactivity, are linked with poor health conditions. People in poor health conditions are more likely to have negative experiences in the health care system and be less satisfied with it [29]. Lower patient satisfaction tends to decrease the probability of seeking health care [30]. Third, lifestyles result in chronic diseases, specifically heart disease, diabetes, and cancer [31]. People with lifestyle behaviors including current smoking, regular drinking, and physical inactivity may suffer from long-term chronic conditions and more than one chronic condition, which often leads to normalization of symptoms and thus inhibits health care seeking [32]. Last, lifestyle factors contribute to productive losses at work and reduced ability to work [33, 34]. People with lifestyle behaviors including current smoking, regular drinking, and physical inactivity may sacrifice some of their leisure time to perform unfinished work. Time constraints may delay some people from seeking health care [35].

Although the present study used a national survey to analyze the lifestyle factors affecting health care-seeking behavior among adults who reported physical discomfort, several limitations should be emphasized. First, the CFPS survey does not collect information on self-medication practice, so this study defined health care-seeking behavior as the seeking of professional help. Previous studies have shown that individuals who perceive themselves to have mild health problems are more likely to self-medicate in China [36, 37]. Second, data were obtained via a survey, and thus the limitations of all self-reported data such as recall bias and social desirability bias also apply here. Finally, although the present study adjusted for a large variety of control variables, it is possible that unknown or unmeasured confounders may explain the current findings.

## Conclusions

This study estimated the effects of current smoking, regular drinking, and physical inactivity on health care-seeking behavior among adults who reported physical discomfort in China. The empirical findings suggested that current smoking, regular drinking, and physical inactivity decreased the probability of seeking health care among adults who reported physical discomfort. Therefore, primary-level care should deliver screening and brief advice programs and pay more attention to those with lifestyle behaviors such as current smoking, regular drinking, and physical inactivity, thus avoiding missed opportunities to treat chronic conditions and detect new diseases early.

## Data Availability

The datasets generated and/or analyzed during the current study are available in the Peking University Open Research Data Platform repository, https://opendata.pku.edu.cn/dataset.xhtml?persistentId=doi:10.18170/DVN/45LCSO.
